# Multimodal Analgesia with Local Wound Infiltration and Intravenous Parecoxib for Thyroidectomy

**DOI:** 10.3390/medicina59050855

**Published:** 2023-04-28

**Authors:** Tz-Ping Gau, Sheng-Hua Wu, Jui-Mei Huang, Wen-Ling Lu, Tzu-Yen Huang, I-Cheng Lu, Che-Wei Wu

**Affiliations:** 1 Department of Anesthesiology, Kaohsiung Medical University Hospital, Kaohsiung Medical University, Kaohsiung 807, Taiwan; u9401066@gap.kmu.edu.tw; 2 Center for Big Data Research, Kaohsiung Medical University, Kaohsiung 807, Taiwan; 3 Department of Information Management, National Sun Yat-sen University, Kaohsiung 804, Taiwan; 4 Department of Anesthesiology, Kaohsiung Municipal Ta-Tung Hospital, Kaohsiung Medical University Hospital, Kaohsiung Medical University, Kaohsiung 801, Taiwan; 5 Faculty of Medicine, College of Medicine, Kaohsiung Medical University, Kaohsiung 807, Taiwan; 6 Department of Anesthesiology, Kaohsiung Municipal Siaogang Hospital, Kaohsiung Medical University Hospital, Kaohsiung Medical University, Kaohsiung 812, Taiwan; 7 Department of Otorhinolaryngology—Head and Neck Surgery, Kaohsiung Medical University Hospital, Kaohsiung Medical University, Kaohsiung 807, Taiwan

**Keywords:** multimodal analgesia, monitored thyroidectomy, local infiltration, lidocaine, parecoxib

## Abstract

*Background and objective*: Adequate postoperative pain control is an important component to enhance recovery. Multimodal analgesia with various pain control techniques has been widely used to alleviate postoperative pain. The use of either wound infiltration or a superficial cervical plexus block has been reported to be effective for pain management after thyroid surgery. The present study evaluated the effect of multimodal analgesia using lidocaine wound infiltration combined with intravenous parecoxib for patients monitored after thyroidectomy. *Materials and Methods:* A total of 101 patients with a multimodal analgesia protocol being monitored after thyroidectomy were enrolled. After the induction of anesthesia, multimodal analgesia was performed through wound infiltration of 1% lidocaine and epinephrine at a ratio of 1:200,000 (5 μg/mL) combined 40 mg intravenous parecoxib before skin excision. Patients were divided into two groups for this retrospective analysis based on the injection dose of lidocaine they received. Patients in Group I (the control, *n* = 52) received a 5 mL injection solution, while those in Group II (the study, *n* = 49) received a 10 mL dosage in a time-sequential manner, in accordance with a previous clinical trial. The primary outcome was measuring postoperative pain intensity at rest, as well as during motion and coughing, which was measured at the postoperative anesthetic care unit (PACU) and on the first day after the operation (POD 1) in the ward. Pain intensity was assessed using a numerical rating scale (NRS). The secondary outcomes were postoperative adverse events including anesthetic-related side effects, as well as airway and pulmonary complications. *Results:* Most of the patients reported no pain or mild pain during the observation period. The patients in Group II had a lower pain intensity during motion than Group I (NRS 1.47 ± 0.89 vs. 1.85 ± 0.96, *p* = 0.043) when measured at the postoperative anesthetic care unit. Pain intensity during coughing was also significantly lower in the study group than in the control group (NRS 1.61 ± 0.95 vs. 1.96 ± 0.79, *p* = 0.049) when measured at the postoperative anesthetic care unit. There were no severe adverse events in either of the groups. Only one patient (1.9%) in Group I experienced temporary vocal palsy. *Conclusions:* The use of lidocaine with an equal volume of intravenous parecoxib provided comparable analgesia with minimal adverse events when monitoring thyroidectomy.

## 1. Introduction

Adequate postoperative pain control is important for enhancing recovery [1,2] and reducing the development of chronic pain [3,4]. Multimodal analgesia combined with various analgesic agents or techniques is widely known to attenuate acute pain after surgery, while reducing the use of opioids, which can lead to a variety of adverse events. In a recent review of non-opioid analgesics for pain management after thyroid surgery, local anesthetics and nonsteroidal anti-inflammatory drugs (NSAIDs) were highly recommended. Acetaminophen, gabapentin, and ketamine have been noted as adequate alternatives [5]. 

Local or regional analgesia plays an important role in multimodal analgesia. The rationale behind locoregional analgesia is that local anesthetics can block nociception transmission peripherally at the surgical site. Both wound infiltration and superficial cervical plexus block have been reported to be effective for pain management after thyroidectomy [6,7]. Hoh SY et al. reported that bilateral superficial cervical plexus block attenuated postoperative pain within 24 h in 35 patients undergoing thyroid surgery. The mean pain score decreased from 2.69 to 1.43, and only 15 of 35 patients required rescue tramadol injection [7]. Both parecoxib and NSAIDs could alleviate pain and inflammation by reducing prostaglandin synthesis by inhibiting the cyclooxygenase-2 (COX-2) enzyme. As a non-opioid analgesic, parecoxib has become widely popular in multimodal analgesia because of its enhanced recovery after surgery (ERAS) [8,9]. 

The thyroid gland, which consists of two interconnected lobes, is one of the biggest endocrine glands found in humans, measuring between 20 to 30 g. The incidence of thyroid lesions in this gland is between 4% and 7%. The majority are asymptomatic and have normal thyroid hormone secretion [10]. Thyroid surgery with intraoperative neural monitoring has become a preferred option for thyroid goiter. The purpose of the present study was to assess the effect of preemptive multimodal analgesia using a combination of lidocaine wound infiltration and intravenous parecoxib in patients being monitored after thyroidectomy. To the best of our knowledge, the combination of local wound infiltration of 1% lidocaine with intravenous parecoxib has not been investigated for pain reduction in thyroidectomy using intraoperative neural monitoring. We hypothesized that a higher infiltration volume (10 mL) of 1% lidocaine might provide better analgesic effects than the conventional volume (5 mL), without creating adverse events.

## 2. Materials and Methods

### 2.1. Design

This retrospective observational research is a secondary analysis of a prior clinical trial registered with ClinicalTrials.gov (NCT 04982185), which evaluated the feasibility of routinely monitoring neuromuscular blockage during when monitoring patients after thyroidectomy. The trial was conducted in a regional teaching hospital in Kaohsiung, Taiwan. The Institutional Review Board of Kaohsiung Medical University Hospital (KMUHIRB-E(I)-20210070) granted ethical approval. By utilizing data from the original trial, the main goal of this retrospective analysis was to investigate the analgesic effects of multimodal analgesia by combining local wound infiltration of 1% lidocaine and intravenous parecoxib in patients being monitored after thyroidectomy. Specifically, the research compared the efficacy of two concentrations that had previously been used. We gathered the medical records of patients with either benign or thyroid cancer undergoing elective thyroidectomy with intraoperative neural monitoring, aged between 20–80 years, enrolled at the American Society of Anesthesiologists [1], with a status of I~III. Patients with the following criteria were excluded from the study: chronic pain with any analgesics, consumption of any painkillers within one day before admission, allergy to NSAID or parecoxib, existing vocal cord palsy, and previous thyroid surgery. The intraoperative neural monitoring setup, thyroidectomy procedures, and anesthesia protocol followed the International Neural Monitoring Study Group Guidelines [11,12,13].

### 2.2. Anesthesia Induction and Maintenance 

During preparation for anesthesia, each patient was placed in a supine position with neck extension, using a donut pad, for their thyroidectomy. Before the induction of anesthesia, standard physiological monitoring (oximetry, electrocardiography, non-invasive blood pressure, and capnography) and anesthesia depth monitoring (bispectral index or response entropy) were established. Neuromuscular transmission was continuously monitored through adductor pollicis muscle stimulation considering the train-of-four (TOF) ratio. General anesthesia was induced with fentanyl (1 µg/kg), lidocaine (1 mg/kg), propofol (2 mg/kg), and rocuronium (0.6 mg/kg). Oral endotracheal tubes with an internal diameter of 7.0- or 7.5-mm were used for females and males, respectively. An experienced anesthesiologist performed tracheal intubation using a video intubating stylet (Trachway, Biotronic Instrument Enterprise Ltd., Tai Chung, Taiwan) when the lowest TOF ratio was noted. The accurate position of each endotracheal tube was confirmed using both auscultation and capnography. Mechanical ventilation was set up to keep the end-tidal CO2 between 35 and 45 mmHg, while anesthesia was maintained with sevoflurane and propofol target-controlled infusion to keep bispectral index or response entropy value between 40 and 60. A bolus of 8 mg ephedrine and rapid infusion of lactated Ringer’s solution was administrated if the mean arterial pressure dropped 20% lower than baseline for longer than two minutes. 

### 2.3. Multimodal Analgesia and Study Protocol 

**Figure 1:**
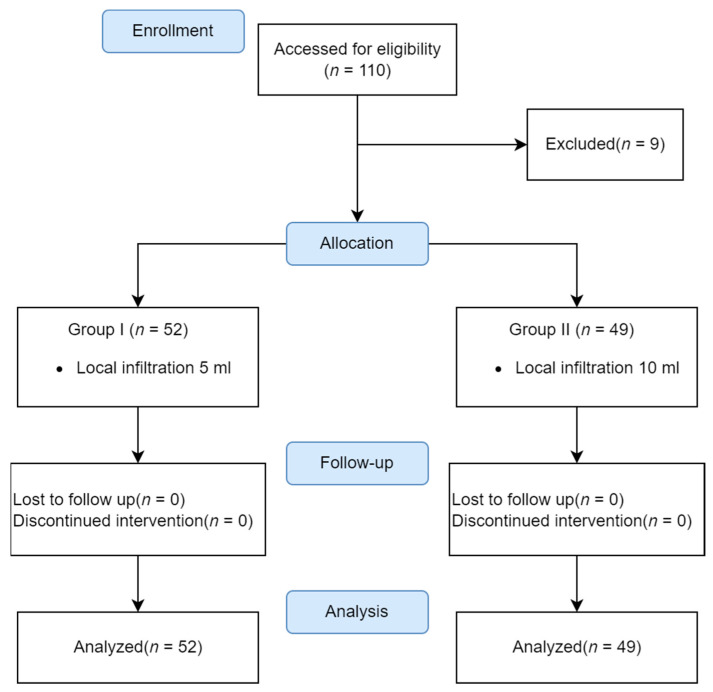
Study flow chart of 101 participants receiving multimodal analgesia.

All patients received preemptive multimodal analgesia before skin incision via (1) local infiltration over the incision line with 1% lidocaine mixed epinephrine at 1:200,000 (5 μg/mL), (2) intravenous parecoxib 40 mg, and (3) a bolus of fentanyl 0.5 μg/kg. To prevent the toxicity associated with the injection of lidocaine, all pharmaceutical dosages were validated to be less than 7 mg/kg, which is the upper limit of systemic toxicity after reducing the rate of absorption using vasoconstrictors such as epinephrine [14]. Then, 5 mg of dexamethasone was injected intravenously in both groups in order to prevent postoperative nausea and vomiting (PONV). In a previous trial, patients received subcutaneous wound infiltration through 5 mL local injection for the first 6 months, which was increased to 10 mL for the following 6 months. In this retrospective study, patients who received the old protocol of 5 mL were assigned to Group I (control), and those who received the new protocol of 10 mL were assigned to Group II (study) based on their time sequence. Figure 1 displays the distribution of patients among the different groups.

Thyroidectomies were performed by the same surgeon following the standardized intraoperative neural monitoring protocol recommended by the international guidelines. According to departmental guidelines, thyroid surgery included total thyroidectomy and total lobectomy. Subtotal thyroidectomy was not performed in everyday practice. Skin incisions were made along previous local infiltration areas on the lower neck, followed by dissection of the central neck. After the skin flap was elevated, two pairs of trans-thyroid cartilage recording electrodes were used to record the electrophysiologic electromyographic (EMG) response that was evoked [15]; they were connected to an NIM response 3.0 monitoring system (Medtronic, Jacksonville, FL). A press monopolar ball-tip probe was used for intraoperative laryngeal nerve stimulation (intensity of 1 to 3 mA, frequency of 4 Hz, duration of 100 µs, and event threshold of 100 µV). Sevoflurane was discontinued after resection of the thyroid and propofol infusion was continued until the end of surgery. Patients were extubated when a TOF ratio > 0.95 and adequate spontaneous ventilation were simultaneously observed. After responding to verbal commands, patients were transferred to the postoperative anesthetic care unit (PACU). 

### 2.4. Outcome Measurements 

Postoperative pain intensity was assessed using the numerical rating scale (NRS) at rest, as well as during motion and coughing. The pain assessment time points were taken before discharge from PACU, on the first day after the operation (POD 1) at the ward, and one week after the operation during the outpatient department (OPD) visit. If a patient suffered from moderate pain (NRS > 3) at PACU, 25 μg of fentanyl was administrated as a rescue analgesic. If a patient had persistent moderate pain (NRS > 3) during their OPD visit, 25 mg of diclofenac was prescribed. Postoperative adverse events included lidocaine toxicity-related transient neurologic symptoms (TNS), PONV, dizziness, pruritus, sore throat, airway obstruction, and postoperative pulmonary complications, with postoperative observations being recorded by a registered nurse anesthetist who was blinded to the group allocation. The outcome of intraoperative neural monitoring was evaluated using vocal palsy (both temporary and permanent). 

### 2.5. Statistic Analysis 

We used an online website (http://powerandsamplesize.com, accessed on 8-11-2022) to calculate the sample size needed in order to show at least a 15% difference in pain intensity (NRS) between groups. The power analysis found that at least 50 patients per group could be used in order to obtain a power of 0.8 with an alpha error of 0.05. Therefore, we enrolled 60 patients per group. Anesthesia data were expressed as mean ± (standard deviation). All data were expressed as mean or number of patients (%). Continuous variables between groups were carried out using Student’s *t*-test, while categorical nominal variables were analyzed using the chi-square test or Fisher’s exact test, as applicable. All statistical tests were two-tailed, and a *p*-value < 0.05 was considered statistically significant. Statistical analysis was performed with R language (version 4.1.3) and RStudio software (Integrated Development Environment for R. Boston, MA, version 2022.07.0+548).

## 3. Results 

In total 101 (26 males and 75 females; aged between 20 and 79 years) completed the study (Figure 1). The physical status and surgical parameters of the patient characteristics did not differ significantly between groups (Table 1). Regarding the surgical indication of 101 patients undergoing monitored thyroidectomy, 41 patients were diagnosed as having benign goiter and 60 patients as thyroid cancer. 

**Figure 2:**
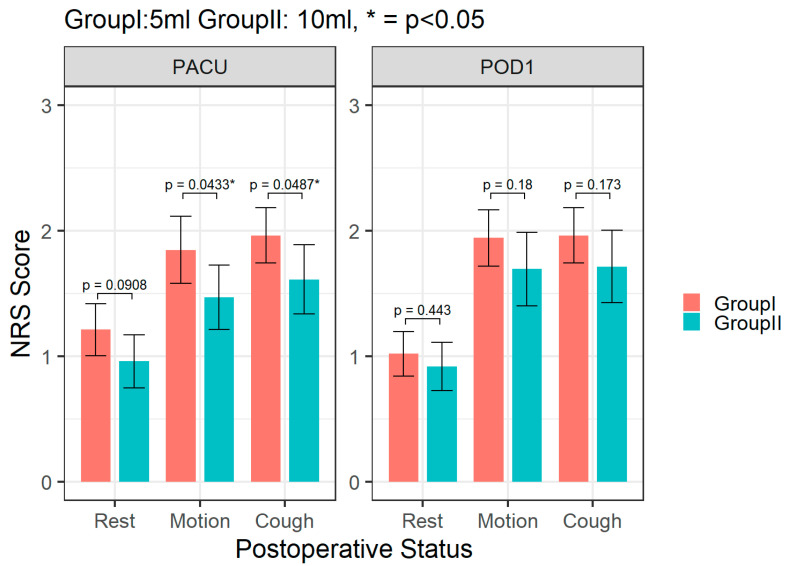
Pain intensity of two groups under various statuses at the postoperative care unit (PACU) and on the first day after the operation (POD 1). Columns show the mean score, error bars are standard deviation. * *p* < 0.05; NRS = numerical rating scale.

**Figure 3:**
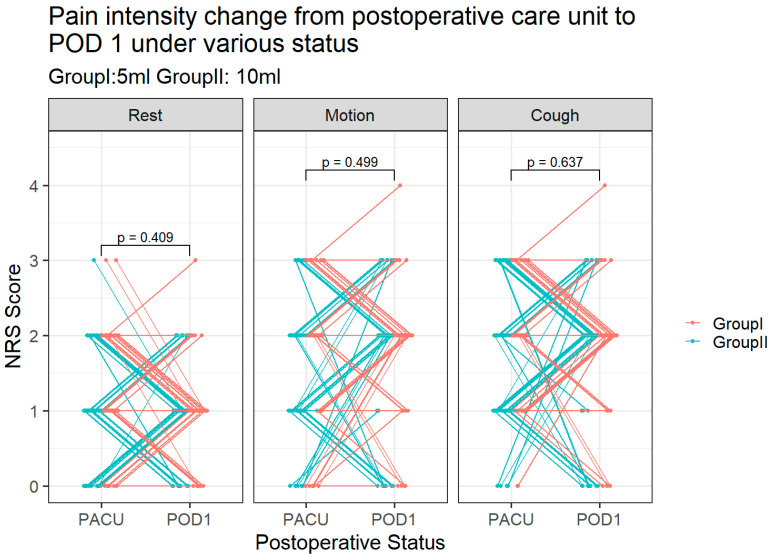
Change in pain scores from PACU to POD 1 under various statuses. Each line represents one case; NRS = numerical rating scale.

Postoperative pain intensity as assessed by NRS is demonstrated in Figure 2 and Table 2. Compared with Group I, the patients in Group II had a lower pain intensity during neck motion than the control group (NRS 1.47± 0.89 vs. 1.85± 0.96, *p* = 0.043) upon discharge from the postoperative anesthetic care unit (Figure 2). Pain intensity during coughing was also significantly lower in Group II than Group I (NRS 1.61± 0.95 vs. 1.96± 0.79, *p* = 0.049) at the same time point (Figure 2). The pain intensity under three statuses did not reveal significant difference between groups at POD 1 (Figure 2). Group II presented less pain intensity than Group I during the OPD visit (Table 2). Figure 3 shows there was no difference between groups considering a change in pain score change from PACU to POD 1. Within the observation period, most of the surgical patients revealed no pain or mild postoperative pain. Only one patient in Group I had moderate pain (NRS = 4) during motion and coughing at POD1 (Figure 3).

As shown in Table 2, the incidence of adverse postoperative events observed in PACU and POD 1 was not significantly different between the two groups. These adverse effects included nausea and vomiting, dizziness, pruritus, sore throat, airway events, and pulmonary complications. This comprehensive study of the potential postoperative complications demonstrates the safety and tolerability of the investigated analgesic regimen and contributes to a more robust view of its overall efficacy in the context of thyroidectomy with intraoperative neural monitoring. In addition, no transient neurologic symptoms associated with lidocaine toxicity were identified in the observation data of both groups of patients in the postoperative care unit.

No instances of permanent vocal palsy were observed in patients undergoing monitored thyroidectomy in either Group I or Group II. One patient in Group I experienced a temporary episode of vocal palsy and spontaneous recovery was noted within 6 weeks postoperatively. A comparison of the frequencies of temporary and permanent vocal palsy between Group I and Group II revealed no significant differences (*p* = 0.3 and 1.0, respectively).

## 4. Discussion 

This study has demonstrated that intravenous parecoxib combined with either 5 or 10 mL 1% lidocaine local wound infiltration was effective for acute pain control after thyroidectomy. Patients receiving 10 mL pre-incision infiltration volume revealed a lower pain intensity during motion on the day of operation and less wound pain one week after the operation. Pain at other time points and all postoperative adverse events were comparable between groups.

Sufficient postoperative pain control is a major component of functional recovery. Both opioid and non-opioid analgesics can be used effectively. With respect to multimodal analgesia for thyroidectomy, an opioid-sparing regimen is mandatory for providing analgesia with minimal opioid adverse events [16]. In a systematic review of seven randomized controlled trials, six trials enrolled multimodal analgesia into ERAS protocols for thyroid and parathyroid surgery [2]. Yip et al. reduced the morphine requirement by up to 72% after thyroid and parathyroid surgery via multimodal analgesia including preoperative oral acetaminophen and gabapentin, intraoperative dexamethasone, bupivacaine local wound infiltration, and postoperative acetaminophen, with opioid rescue used only when necessary [17]. 

Locoregional analgesia with local anesthetics is highly recommended as a mandatory component of multimodal analgesia for most ERAS protocols [8,9]. Local anesthetics block nerve conduction, inhibit central sensitization, and suppress the release of inflammatory mediators. These pharmacologic characteristics result in an analgesic effect and decreased pain intensity. Both local wound infiltration and superficial cervical plexus block have been reported to control acute pain after thyroidectomy. In fact, controversy exists regarding the analgesic efficacy of locoregional block as an independent factor in thyroidectomy [5,18,19]. Our departmental protocol of local wound infiltration is preemptive and is conducted before skin incisions to reduce noxious stimulation and blood loss. Miu et al. reported that local wound infiltration with 10 mL of 0.75% ropivacaine after thyroid surgery did not provide a lower pain score or morphine sparing when compared with the placebo [19]. In a meta-analysis, the effect of bilateral superficial cervical plexus block on pain reduction was considered insufficient to be clinically relevant and still resulted in a significant pain score 24 h after surgery. [18].

The purpose of multimodal analgesia is to obtain better outcomes (i.e., low pain score) rather than to validate specific techniques or agents. We found that the combination of local wound infiltration and intravenous parecoxib provided adequate pain control and was well-tolerated. In comparison with the cervical plexus block, local wound infiltration was safe and easy to perform without ultrasound guidance. Moreover, local wound infiltration could also avoid possible adverse events related to cervical plexus block, such as phrenic nerve palsy, inadvertent vessel puncture, and local anesthetic intoxication [20,21]. 

Parecoxib sodium is the only intravenous selective cyclooxygenase-2 (COX-2) inhibitor to provide analgesia and anti-inflammation effects. After administering parecoxib sodium, it is rapidly converted to valdecoxib and reduces prostaglandin synthesis [22]. Except for its contraindications, COX-2 inhibitors are highly recommended part of multimodal analgesia for opioid-sparing and decreased opioid-related side effects [23]; moreover, parecoxib exerts preemptive analgesia without interfering with the coagulation function. In a minor surgery such as micro-laryngeal surgery, parecoxib might be comparable to morphine considering postoperative pain control, with less adverse events [24]. However, in a major surgery, i.e., total knee replacement, patient-controlled analgesia through preemptive parecoxib in combination with morphine showed better pain relief than morphine alone in PACU [25]. Meanwhile, in previous research, for patients experiencing pain after thyroid surgery, the combination of intravenous (IV) paracetamol either with intramuscular (IM) pethidine or IV parecoxib showed better analgesia than IV paracetamol alone, from 45 min after transfer to the surgical ward until 24 h after surgery [26]. All the aforementioned studies demonstrate that the concurrent use of parecoxib as part of a successful clinical pain management approach is backed by evidence. However, the analgesic impact of these medicines did not last until OPD follow-up, and there was no change in pain score from PACU to the POD 1 under various conditions.

There were several limitations in this retrospective study. Firstly, the design was non-randomized because it aimed to review existing clinical analgesic regimens, although the patient characteristics, disease, and surgical profiles were comparable between the two groups. Secondly, there could be a selection bias due to the patients being selected and surgical procedures being performed by a single surgeon in a healthcare system. All thyroid surgeries were performed using an intraoperative neural monitoring system; in Taiwan, this is a self-payment regimen. The study only enrolled patients who could afford to be monitored after thyroidectomy; therefore, they could be of a higher socioeconomic status. Furthermore, both total thyroidectomy and subtotal thyroidectomy are major procedures for thyroid tumors. Total thyroidectomy does not increase complications, such as hematoma or recurrent laryngeal nerve, compared to subtotal thyroidectomy [24]. The surgeon routinely performed total thyroidectomy without leaving thyroid remnant after identification of recurrent laryngeal nerve, according to the institutional standardized IONM protocol. Finally, any practice of locoregional analgesia requires a well-communicated team. Lidocaine toxicity is not only dictated by the total dosage, but also by the rate of absorption; thus, further precautions must be taken to avoid direct toxic nerve effects from the high concentration of lidocaine when implementing our multimodal analgesia protocol and interpreting the study outcomes.

## 5. Conclusions

Multimodal analgesia using a combination of 1% lidocaine local wound infiltration with 40 mg parecoxib intravenous injection provided effective postoperative pain control with low adverse events when monitoring after thyroidectomy. The higher local wound infiltration volume of 10 mL 1% lidocaine was superior to 5 mL considering pain score on the day of operation and one week after the thyroidectomy. To minimize the risk of local toxicity from anesthetics, caution should be taken when selecting the injection technique and total dose of local anesthetics. Local infiltration might be as effective as superficial cervical plexus block, with minimal local anesthetics toxicity events. Further study is needed to determine whether local anesthetics with a longer duration (i.e., ropivacaine and bupivacaine) are more effective than lidocaine for postoperative pain control after thyroidectomy. 

## Figures and Tables

**Table 1:** Patient characteristics.

	Group I (*n* = 52)	Group II (*n* = 49)	*p*
Female/Male (*n*)	38/14	37/12	0.959
Age (*y/o*)	48.9 ± 12.8	53.0 ± 12.8	0.109
ASA status ^1^ (*n*)			0.195
I	3 (52.2%)	1 (2.0%)	
II	45 (43.5%)	39 (79.6%)	
III	4 (43.5%)	9 (18.4%)	
Operation (*n*)			0.169
Total thyroidectomy	27 (51.9%)	33 (67.3%)	
Total lobectomy	25 (48.1%)	16 (32.7%)	
Operation time (minutes)	72.9 ± 16.3	75.2 ± 17.2	0.508
Nerve at risk (*n*)	79	82	
Temporary vocal palsy	1 (1.9%)	0 (0%)	0.306
Permanent vocal palsy	0 (0%)	0 (0%)	1

^1^ ASA = American Society of Anesthesiologists physical status classification.

**Table 2:** Postoperative adverse events.

	Group I (*n* = 52)	Group II (*n* = 49)	*p*
Rescue analgesics at PACU ^1^	2 (3.8 %)	0 (0 %)	0.166
Pain intensity at OPD ^2^			0.026
None (NRS ^3^ = 0)	27 (51.9%)	38 (77.6%)	
Mild (NRS = 1~3)	23 (44.2%)	10 (20.4%)	
Moderate (NRS > 3)	2 (3.9 %)	1 (2.0 %)	
Nausea/vomiting			
at PACU (*n*)	13 (25%)	12 (24.5%)	0.952
at POD 1 ^4^ (*n*)	1 (1.9%)	1 (2.0%)	0.966
Dizziness			
at PACU (*n*)	14 (26.9%)	14 (28.6%)	0.853
at POD 1 (*n*)	3 (5.8%)	2 (4.1%)	0.94
Pruritus (*n*)	0 (0%)	0 (0%)	1
Sore throat (*n*)	9 (17.3%)	12 (24.5%)	0.374
Airway events ^5^ (*n*)	0 (0%)	0 (0%)	1
Pulmonary complications (*n*)	0 (0%)	0 (0%)	1

^1^ PACU = postoperative anesthetic care unit; ^2^ OPD = outpatient department; ^3^ NRS = numerical rating scale; ^4^ POD 1 = first day after operation; ^5^ Airway events = airway obstruction or hematoma after surgery.

## Data Availability

The data of the current study are available from the corresponding author upon request.
